# Prevalence and longitudinal development of sarcopenic obesity in an Asian obese population: a large bioelectrical impedance analysis study

**DOI:** 10.3389/fnut.2026.1828960

**Published:** 2026-06-02

**Authors:** Guo Hou Loo, Farhana Raduan, Zunaidah Abu Samah, Nik Ritza Kosai

**Affiliations:** 1 Upper GI and Metabolic Surgery Unit, Department of Surgery, Faculty of Medicine, Universiti Kebangsaan Malaysia, Cheras, Malaysia; 2 Physiotherapy Unit, Department of Medical Rehabilitation Service, Hospital Canselor Tuanku Muhriz, Universiti Kebangsaan Malaysia (UKM), Cheras, Malaysia

**Keywords:** bioelectrical impedance analysis, body composition, ESPEN/EASO, longitudinal reclassification, Malaysia, muscle–fat ratio, sarcopenic obesity

## Abstract

**Background:**

Sarcopenic obesity (SO), the coexistence of excess adiposity and reduced relative muscle mass, has metabolic and functional implications. Data in Asian populations remain limited.

**Objectives:**

To determine SO prevalence using two diagnostic frameworks, identify predictors of SO while avoiding circular inference, and examine longitudinal reclassification in initially non-sarcopenic obese individuals.

**Methods:**

Retrospective cohort study of 2,410 adults with BMI ≥ 25 kg/m^2^ undergoing bioelectrical impedance analysis (BIA) at a Malaysian tertiary centre (2017–2025). SO was defined using AWGS 2019 skeletal muscle mass index (SMI) thresholds and an adapted ESPEN/EASO-based definition with sex-specific body fat percentage and population-derived lowest-quartile muscle–fat ratio (MFR) thresholds. Multivariable logistic regression, Kaplan–Meier and Cox proportional hazards analyses were applied to the longitudinal cohort.

**Results:**

SO prevalence was 0.12% (AWGS) and 25.02% (adapted ESPEN/EASO), with equivalent prevalence across sexes (females: 25.02%; males: 25.03%; *p* = 1.00). The SO group had significantly higher BMI, body fat percentage, and SMI. In multivariable analysis (MFR excluded to avoid circular inference), higher BMI (OR: 1.248; 95% CI: 1.204–1.304), male sex (OR: 1.757; 95% CI: 1.381–2.321), and older age (OR: 1.277; 95% CI: 1.136–1.435) were statistically associated with SO in this conditional model; these associations require cautious interpretation given conceptual overlap between BMI and the adiposity component of the definition. Of 742 initially non-sarcopenic individuals, 47 (6.3%) were reclassified during follow-up (6.5% at 6 months, 12.0% at 12 months); in Cox proportional hazards regression, baseline BMI was the only independent predictor of time to reclassification (HR: 1.098; 95% CI: 1.019–1.195).

**Conclusion:**

SO affects approximately one in four obese Malaysian adults under the present body composition-based criteria (sex-specific body fat percentage and population-derived lowest-quartile MFR thresholds); this estimate is contingent on the operational definition and is not an externally validated epidemiological prevalence. MFR and BMI are the most clinically informative metrics, but MFR must not be simultaneously used to define and predict SO. Longitudinal BIA monitoring is warranted in obese populations, as a measurable proportion is reclassified within 12 months.

## Introduction

Obesity remains one of the most significant global public health challenges of the 21st century, with particularly rapid escalation in the Asia-Pacific region (1). Malaysia has one of the highest obesity prevalences in Southeast Asia, with recent national surveys reporting that over half of the adult population is living with overweight or obesity (2). Importantly, Asian populations experience obesity-related metabolic and cardiovascular risks at lower BMI thresholds compared with Western populations, prompting the adoption of region-specific BMI cut-offs (3–5). Correspondingly, assessment of basal metabolic rate and body composition using standardised methods is increasingly recognised as essential for accurate metabolic risk characterisation in overweight and obese individuals (6).

While the adverse health consequences of obesity are well established, increasing attention has been directed toward sarcopenic obesity (SO), a complex phenotype defined by the coexistence of excess adiposity and impaired relative muscle mass (7, 8). Sarcopenic obesity is associated with compounded risks of metabolic dysfunction, physical disability, frailty, and increased cardiovascular morbidity and mortality (8, 9). Its pathophysiology involves a dysregulated interplay between adipose tissue expansion, chronic low-grade inflammation, insulin resistance, hormonal alterations, physical inactivity, and accelerated muscle catabolism (9, 10). Notably, SO is increasingly recognised not only among older adults but also in middle-aged individuals, particularly in Asian populations where differences in body composition and fat distribution are prominent (10, 11).

Estimating the true burden of SO remains challenging owing to heterogeneity in diagnostic definitions. Traditional approaches have relied on skeletal muscle mass index (SMI), derived from appendicular skeletal muscle mass adjusted for height, using AWGS 2019 region-specific cut-offs (10). However, in individuals with obesity, absolute muscle mass and SMI may be preserved or elevated due to increased total body weight, potentially masking relative muscle deficiency (12, 13). Contemporary frameworks, including the ESPEN/EASO 2022 consensus, emphasise the imbalance between muscle and fat compartments rather than absolute muscle quantity, incorporating ratio-based and fat-adjusted indices that demonstrate greater sensitivity in obese cohorts (7, 14, 15).

Bioelectrical impedance analysis (BIA) provides a practical, non-invasive, and scalable method for assessing body composition in clinical settings, with acceptable validity for estimating fat and muscle compartments (12, 13). Data on SO prevalence, associated risk factors, and longitudinal development in Southeast Asian obese populations remain limited. Most existing studies are cross-sectional, originate from Western cohorts, or apply a single diagnostic framework (14, 15).

Accordingly, this study aimed to: (1) determine SO prevalence using both SMI-based and adapted ESPEN/EASO-based criteria; (2) identify independent predictors of SO using a methodologically sound regression model that avoids circular inference; and (3) examine the longitudinal reclassification to SO among initially non-sarcopenic obese individuals, applying Kaplan–Meier and Cox proportional hazards analyses to account for heterogeneous follow-up intervals.

## Methodology

### Study design and setting

A retrospective observational cohort study was conducted at Hospital Canselor Tuanku Muhriz (HCTM), a tertiary academic centre affiliated with Universiti Kebangsaan Malaysia (UKM). BIA data were obtained from routine clinical assessments conducted between January 2017 and April 2025. Ethical approval was granted by the UKM Research Ethics Committee (Reference No. FF-2025-458); informed consent was waived given the retrospective design and use of anonymised data. The study was conducted in accordance with the Declaration of Helsinki (16).

### Study population

Adult patients aged ≥18 years with BMI ≥ 25 kg/m^2^ were eligible, consistent with WHO Asia-Pacific obesity criteria (4, 5). Patients were excluded if key variables (age, sex, height, weight, skeletal muscle mass, or fat mass) were missing. For cross-sectional analyses, only the most recent BIA record per patient was retained. For longitudinal analyses, all serial records were preserved.

### Body composition assessment

Body composition was assessed using the InBody 370 multi-frequency BIA analyser (Biospace Co., Ltd., Seoul, Korea) per standard institutional protocol. Parameters extracted included body weight, height, BMI, fat mass, body fat percentage (%), skeletal muscle mass (SMM, kg), SMI (kg/m^2^), and muscle–fat ratio (MFR = SMM/fat mass).

### Definition of sarcopenic obesity

AWGS 2019 (SMI-based): Low muscle mass was defined as SMI < 7.0 kg/m^2^ (males) and <5.7 kg/m^2^ (females) (10).

Adapted ESPEN/EASO-based definition: Given the absence of externally validated normative MFR cut-offs for Asian obese populations, SO was operationalised as the coexistence of: (i) elevated body fat percentage (≥30% males; ≥40% females) and (ii) MFR in the sex-specific lowest quartile within the study cohort (≤0.740 males; ≤0.501 females). This approach follows the ESPEN/EASO recommendation to derive population-specific relative muscle mass thresholds when external normative data are unavailable (7). It is acknowledged that population-derived Q1 thresholds structurally assign approximately 25% of each sex to the low-MFR category; the resulting prevalence estimates should therefore be interpreted as reflecting the proportion of the obese population meeting a relative compositional threshold, not an externally validated absolute diagnostic criterion.

### Statistical analysis

Continuous variables are reported as means with standard deviations (SD). Group comparisons used Welch’s independent-samples t-tests. Chi-squared test was used to compare SO prevalence between sexes. Multivariable logistic regression with bootstrapped confidence intervals (500 resamples) was performed to identify independent predictors of SO; to avoid circular inference, MFR was excluded from the regression model (see Limitations). The final model incorporated age (per decade), sex, and BMI. For longitudinal analysis, Kaplan–Meier survival analysis estimated cumulative probability of SO reclassification over time, with log-rank test for group comparisons. A Cox proportional hazards (Cox PH) regression model assessed the association of baseline characteristics (age, BMI, body fat percentage, sex) with time to first observed reclassification to SO; participants without reclassification were censored at their last available follow-up. Given heterogeneous follow-up intervals, 95% confidence intervals for hazard ratios were obtained by bootstrap resampling (500 replicates) to provide stable interval estimates. A two-sided *p* < 0.05 was considered statistically significant. All analyses were performed using IBM SPSS Statistics version 29.0.

## Results

### Patient characteristics

After deduplication and exclusion of incomplete records from 8,252 raw entries, 2,410 individuals were included. Mean age was 45.8 years (SD: 13.6); 58.9% were female. Mean BMI was 35.5 kg/m^2^ (SD: 9.6); mean body fat percentage 43.3% (SD: 9.1). Overall, 33.0% had BMI 25.0–29.9 kg/m^2^ and 67.0% had BMI ≥ 30 kg/m^2^.

### Prevalence of sarcopenic obesity

Using AWGS SMI criteria, SO was identified in 3 individuals (0.12%). By the adapted ESPEN/EASO-based definition, SO was present in 603 individuals (25.02%). Sex-specific MFR thresholds were ≤0.740 for males and ≤0.501 for females. Notably, SO prevalence was equivalent across sexes (females: 25.02%, *n* = 355/1419; males: 25.03%, *n* = 248/991; chi-squared, *p* = 1.00), an expected consequence of applying sex-specific Q1 thresholds independently within each sex. The 209-fold discrepancy between AWGS and adapted ESPEN/EASO estimates reflects the fundamental inadequacy of height-adjusted absolute muscle mass indices in obese populations.

### Body composition comparison: SO vs. non-SO groups

Table 1 summarises body composition differences. The SO group exhibited significantly higher BMI (44.64 ± 9.23 vs. 32.49 ± 7.50 kg/m^2^; *p* < 0.001) and body fat percentage (52.00 ± 3.77% vs. 40.35 ± 8.45%; p < 0.001), and significantly lower MFR (0.51 ± 0.09 vs. 0.95 ± 1.21; *p* < 0.001). Mean age was modestly lower in the SO group (44.71 ± 13.50 vs. 46.16 ± 13.59 years; *p* = 0.023). Skeletal muscle mass was significantly higher in the SO group (30.66 ± 8.17 vs. 27.52 ± 6.43 kg; *p* < 0.001), as was SMI (11.76 ± 2.39 vs. 10.56 ± 4.04 kg/m^2^; *p* < 0.001). This paradoxical elevation of SMM and SMI in the SO group, individuals classified as having compositional sarcopenic obesity, reflects weight-driven inflation of absolute and height-normalised muscle mass in severely obese individuals. This finding directly demonstrates why SMI-based criteria virtually never identify SO in obese cohorts: the definition calibrated for non-obese populations cannot detect the relative muscle-to-fat imbalance that characterises SO.

**Table 1:** Body composition: sarcopenic vs. non-sarcopenic obesity groups (adapted ESPEN/EASO).

Parameters	Sarcopenic obesity (*n* = 603)	Non-sarcopenic obesity (*n* = 1807)	t	*p*
Age, years - mean (SD)	44.71 (13.50)	46.16 (13.59)	−2.272	0.023
BMI, kg/m^2^ - mean (SD)	44.64 (9.23)	32.49 (7.50)	29.285	<0.001
Skeletal muscle mass, kg - mean (SD)	30.66 (8.17)	27.52 (6.43)	8.606	<0.001
Muscle-fat ratio - mean (SD)	0.51 (0.09)	0.95 (1.21)	−15.075	<0.001
Body fat, % - mean (SD)	52.00 (3.77)	40.35 (8.45)	46.416	<0.001
SMI, kg/m^2^ - mean (SD)	11.76 (2.39)	10.56 (4.04)	8.828	<0.001

Data: mean (SD). SO, sarcopenic obesity; BMI, body mass index; SMI, skeletal muscle mass index. All p-values reported to three decimal places or as <0.001.

### Sex-specific differences in body composition

Table 2 presents sex-stratified data. Females had significantly higher BMI, body fat percentage, and lower MFR than males, despite males having greater absolute body weight and SMM. Males were significantly older. Despite these compositional differences, SO prevalence was statistically equivalent (25.02% females vs. 25.03% males; *p* = 1.00), reflecting methodologically appropriate sex-specific thresholding. The higher female compositional risk profile (elevated adiposity, lower MFR) is consistent with sex-specific hormonal influences on fat distribution and muscle mass, including the effects of oestrogen decline during the perimenopausal transition, increased visceral adiposity, and androgen-related skeletal muscle protection in males (11, 17, 18). Despite equivalent final prevalence, clinically, females operating with systematically higher adiposity and lower MFR relative to males are compositionally closer to the SO threshold at any given BMI, warranting targeted monitoring.

**Table 2:** Sex-specific body composition and sarcopenic obesity prevalence.

Parameters	Female (*n* = 1,419)	Male (*n* = 991)	t	*p*
Age, years	44.17 (12.63)	48.12 (14.52)	−6.910	<0.001
Body weight, kg	89.52 (21.76)	96.14 (27.50)	−6.322	<0.001
BMI, kg/m^2^	36.55 (8.85)	34.07 (10.29)	6.152	<0.001
Skeletal muscle mass, kg	25.13 (5.19)	32.86 (6.82)	−30.096	<0.001
Muscle–fat ratio	0.67 (1.04)	1.09 (1.06)	−9.670	<0.001
Body fat, %	47.62 (6.17)	37.02 (8.95)	32.303	<0.001
Non-SO, *n*	1,064	743		
SO, *n*	355	248		
SO prevalence, %	25.02%	25.03%		p = 1.00

Data: mean (SD). SO, sarcopenic obesity. *p* < 0.05 considered statistically significant.

### Independent predictors of sarcopenic obesity

To avoid circular inference since MFR is both a component of the SO definition and a candidate predictor, MFR was excluded from the logistic regression model. The final model incorporated age (per decade), sex, and BMI (Table 3). Higher BMI was the strongest statistical correlate of SO (OR: 1.248; 95% CI: 1.204–1.304; *p* < 0.001), indicating that each unit increase in BMI was associated with a 24.8% increase in odds of SO within the regression framework. This estimate should be interpreted with caution: BMI is mathematically and biologically related to body fat percentage, which forms a core component of the operational SO definition. The observed BMI–SO association therefore partly reflects this conceptual overlap rather than representing a wholly independent aetiological signal, and BMI is best regarded as a clinically useful screening surrogate for the underlying compositional phenotype rather than as a causally independent predictor. Male sex was also significantly associated with SO in the conditional model (OR: 1.757; 95% CI: 1.381–2.321; *p* < 0.001). This finding is, on its face, counterintuitive given that the unadjusted sex-specific prevalence was virtually identical (25.02% females vs. 25.03% males), an expected consequence of applying sex-specific Q1 MFR thresholds within each sex. The apparent discrepancy reflects the conditional nature of the regression: at any given BMI and age, males in this cohort were more likely to meet the operational SO criteria than females, because the male MFR threshold (≤0.740) sits at a higher absolute value than the female threshold (≤0.501) and because male body composition at equivalent BMI carries a different fat–muscle distribution. The marginal (population-level) prevalence and the conditional (model-adjusted) odds therefore answer different questions and are not inconsistent. Accordingly, the sex term should be interpreted as a conditional model coefficient rather than as evidence that male sex is an independent aetiological risk factor for SO at the population level. Older age per decade was also significantly associated with SO (OR: 1.277; 95% CI: 1.136–1.435; *p* < 0.001). Taken together, these regression results identify BMI as the strongest statistical correlate of the body composition-based SO phenotype within this conditional model; however, given the partial conceptual overlap between BMI and the adiposity component of the SO definition, BMI is more appropriately framed as a clinically accessible screening surrogate than as a wholly independent determinant.

**Table 3:** Multivariable logistic regression for predictors of sarcopenic obesity (MFR excluded).

No.	Variable	Odds ratio	95% CI lower	95% CI upper
1	Age (per decade)	1.277	1.136	1.435
2	Sex (Male)	1.757	1.381	2.321
3	BMI (per kg/m^2^)	1.248	1.204	1.304

Bootstrap 500 resamples. Reference: female sex. age expressed per decade. MFR excluded to avoid circular inference (see methods). OR, odds ratio; CI, confidence interval.

### Longitudinal reclassification analysis

Among the 1,017 patients with serial BIA measurements, 742 were non-sarcopenic at their baseline assessment. Of these, 47 (6.3%) were subsequently reclassified as SO. Given the retrospective design, irregular follow-up intervals, and absence of predefined observation endpoints, these findings represent observed body composition reclassification rather than formal disease incidence.

**Figure 1:**
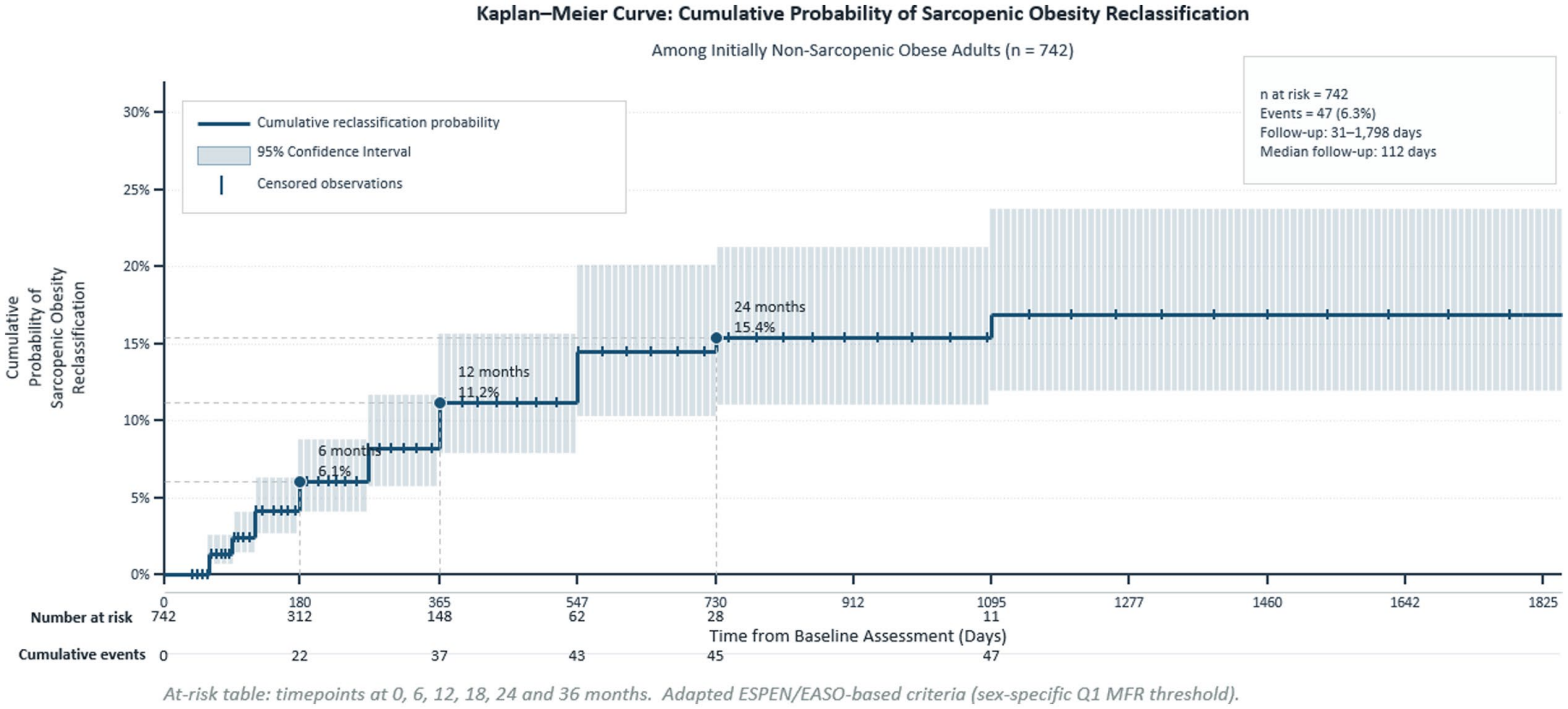
Kaplan–Meier curve showing the cumulative probability of sarcopenic obesity (SO) reclassification over time among 742 initially non-sarcopenic obese adults with serial BIA measurements. Vertical tick marks represent censored observations. Shaded region and dashed lines indicate 95% confidence interval. BIA, bioelectrical impedance analysis; MFR, muscle–fat ratio; SO, sarcopenic obesity.

Kaplan–Meier analysis estimated cumulative SO reclassification probability of 2.7% at 90 days, 6.5% at 180 days, 12.0% at 365 days, and 17.0% at 730 days (Figure 1). Baseline adiposity was the dominant predictor of reclassification: progressors had significantly higher baseline body fat percentage (46.17 ± 5.97% vs. 40.87 ± 9.13%; *p* < 0.001) and BMI (37.08 ± 6.58 vs. 32.72 ± 5.77 kg/m^2^; *p* < 0.001). Baseline age did not differ significantly between groups (42.57 ± 13.17 vs. 45.23 ± 12.65 years; *p* = 0.186).

Cox proportional hazards regression (Table 4) identified baseline BMI as the only independent predictor of time to reclassification to SO (HR: 1.098; 95% CI: 1.019–1.195; *p* = 0.012). Each 1 kg/m^2^ increase in baseline BMI was associated with an approximately 10% increase in the hazard of reclassification. Baseline body fat percentage was not an independent predictor in the fully adjusted model (HR: 1.059; 95% CI: 0.962–1.185; *p* = 0.249), reflecting its strong correlation with BMI in the obese cohort (r = 0.61 within each sex); in single-predictor sensitivity analyses, body fat percentage was significant when entered alone (HR: 1.151; 95% CI: 1.073–1.271; *p* < 0.001), and its loss of independent significance in the full model therefore reflects collinearity with BMI rather than absence of an adiposity–reclassification relationship. Age and sex were not independent predictors of time to reclassification, indicating that adiposity rather than chronological age or sex drives compositional reclassification in this obese cohort.

**Table 4:** Cox proportional hazards regression: predictors of time to sarcopenic obesity reclassification.

No.	Baseline variable	Hazard ratio*	95% CI lower	95% CI upper
1	Age (years)	1.008	0.976	1.039
2	BMI (kg/m^2^)	1.098	1.019	1.195
3	Body fat percentage (%)	1.059	0.962	1.185
4	Sex (male)	1.979	0.470	8.307

*Hazard ratios were estimated from a Cox proportional hazards regression model with time to first observed reclassification to sarcopenic obesity as the time-to-event outcome; participants without reclassification were censored at their last available follow-up. Confidence intervals were derived using bootstrap resampling (500 replicates) to provide stable interval estimates given heterogeneous follow-up intervals. HR, hazard ratio; CI, confidence interval.

## Discussion

In this large retrospective cohort of adults with obesity undergoing routine BIA, we observed substantial variation in SO prevalence depending on the diagnostic framework applied: 0.12% by AWGS SMI criteria versus 25.02% by the adapted ESPEN/EASO-based definition. This 209-fold discrepancy directly demonstrates the unsuitability of absolute height-adjusted muscle mass indices for SO diagnosis in obese populations, a finding consistent with reports from other obese Asian cohorts (12–15).

The low AWGS prevalence reflects the well-documented confounding effect of obesity on SMI: obese individuals carry higher absolute muscle mass due to the mechanical demands of greater body weight, resulting in elevated SMI that masks relative muscle-to-fat imbalance (12, 13). The paradoxically higher SMM and SMI observed in the SO group compared with non-SO individuals directly illustrate this phenomenon, confirming that absolute muscle mass indices should not be used in isolation for SO screening in clinical obesity settings.

The adapted ESPEN/EASO-based definition, incorporating sex-specific body fat percentage and population-derived Q1 MFR thresholds, addresses this limitation by evaluating the compositional imbalance between muscle and fat compartments. This approach is consistent with the ESPEN/EASO consensus recommendation to use relative muscle mass metrics in populations where absolute normative thresholds are unavailable (7). However, it is acknowledged that population-derived Q1 thresholds inherently assign the lowest-quartile compositional phenotype in each sex to the low-MFR category, producing a prevalence that is partly a function of the threshold methodology. These estimates should therefore be interpreted as characterising the relative compositional burden of SO within this population, rather than as externally validated epidemiological estimates. Future studies should prioritise establishing externally validated normative MFR thresholds for Asian obese adults to address this limitation.

Similarly, since MFR constituted a component of the SO definition, including it as a predictor in the multivariable regression model would introduce circular inference. The revised model, incorporating age, sex, and BMI as predictors, avoids this most direct form of methodological concern and demonstrates that BMI is the strongest statistical correlate of the body composition-based SO phenotype in this conditional model (OR: 1.248 per kg/m^2^). The interpretation of BMI as an “independent predictor” nonetheless requires caution. Although MFR was excluded from the model to avoid the most overt form of circularity, BMI remains mathematically and biologically related to body fat percentage, which itself forms a core component of the operational SO definition. The BMI–SO association therefore partly reflects this conceptual overlap rather than representing a wholly independent aetiological signal. The corresponding sex coefficient (OR: 1.757 for males) is similarly best understood as a conditional model parameter rather than as evidence of an independent population-level risk: the marginal sex-specific prevalence is, by design of the sex-specific thresholds, virtually identical (25.02% vs. 25.03%), and the regression coefficient simply reflects how, at equivalent BMI and age, males in this cohort were more likely to meet the operational criteria. The model results are therefore most appropriately framed as identifying clinically accessible markers (BMI, age) that flag obese patients likely to meet the body composition-based SO definition, rather than as identifying causally independent risk factors. This finding has clinical relevance: BMI is universally available, making it a practical first-line screening tool to identify obese patients at elevated SO risk who should proceed to full BIA assessment.

The equivalent SO prevalence across sexes (25.02% females, 25.03% males) is a methodologically expected outcome of applying sex-specific Q1 thresholds independently within each sex. However, important sex differences in compositional risk profiles are evident: females have significantly higher adiposity and lower MFR than males at comparable BMI levels. This difference reflects well-established biological mechanisms, including oestrogen-mediated fat redistribution toward subcutaneous and visceral depots, relative androgen deficiency in females, and the accelerated loss of lean mass and protective muscle function associated with the menopausal transition (17, 18). Postmenopausal oestrogen decline promotes adipogenesis and impairs myogenesis via IGF-1 pathway suppression and increased myostatin expression, while simultaneously redistributing fat from peripheral to visceral depots (18, 19). These hormonal effects place female obese patients at an inherently higher compositional risk of occupying the high-fat, low-MFR phenotype characteristic of SO, even though sex-specific thresholding equalises the final prevalence. Targeted monitoring of MFR trajectories in female obese patients, particularly around the perimenopausal transition is therefore warranted.

The use of population-derived MFR cut-offs, while methodologically transparent, raises valid questions about generalisability (7, 14). The Q1 MFR thresholds derived in this study (≤0.740 males; ≤0.501 females) are specific to this Malaysian obese clinical cohort and may not be directly transferable to other obese populations with different body composition profiles. Comparable population-derived approaches have been applied in the Singaporean Yishun Study and other Asian body composition cohorts, where they demonstrated discriminative validity against adverse health outcomes (15). Nevertheless, the establishment of external reference MFR thresholds derived from large multi-ethnic Asian prospective cohorts with functional outcome data represents a priority for the field.

The longitudinal component of this study identified that 6.3% of initially non-sarcopenic obese patients were reclassified as SO during follow-up, with cumulative reclassification probabilities of 6.5% at 6 months and 12.0% at 12 months by Kaplan–Meier analysis. Cox proportional hazards regression identified baseline BMI as the only independent predictor of time to reclassification (HR: 1.098; 95% CI: 1.019–1.195), corresponding to an approximate 10% increase in the hazard of reclassification per 1 kg/m^2^ increase in baseline BMI. Baseline body fat percentage was significant when entered alone alongside age and sex (HR: 1.151; 95% CI: 1.073–1.271) but lost independent significance once BMI was added to the model (HR: 1.059; 95% CI: 0.962–1.185), consistent with the strong collinearity between BMI and body fat percentage in obese cohorts (r = 0.61 within each sex in our data). BMI and body fat percentage therefore capture overlapping information about adiposity, and BMI, being readily measurable in routine practice, serves as an efficient surrogate for the underlying compositional risk. Baseline age and sex were not independent predictors, confirming that compositional adiposity rather than chronological ageing or sex drives SO reclassification in this working-age obese cohort. These findings underscore the importance of periodic BIA monitoring in obese clinical populations to detect compositional changes before they manifest as overt functional impairment, and they support BMI as a practical first-line trigger for full BIA reassessment. Once identified, patients meeting the body composition-based SO criteria may benefit from combined dietary and resistance-exercise interventions tailored to Asian populations (22).

Several limitations warrant consideration. First, metabolic and inflammatory biomarkers including HbA1c, HOMA-IR, and hs-CRP, were not available in this retrospective dataset. The pathophysiology of SO is tightly linked to insulin resistance and chronic low-grade inflammation; incorporating these parameters in future prospective studies would substantially enrich the characterisation of the SO phenotype and its metabolic correlates. Second, BIA is less precise than DEXA or MRI for body composition assessment; accuracy may be affected by hydration status and extreme adiposity. Third, muscle function assessments such as handgrip strength and gait speed were unavailable, precluding confirmation of functional sarcopenia as recommended by contemporary guidelines (7, 10). The findings accordingly reflect a body composition-based SO phenotype rather than fully defined sarcopenic obesity with functional impairment. Fourth, the MFR Q1 thresholds are population-specific and may have limited generalisability, as discussed above. Also, the retrospective design precludes control for treatment exposures, dietary intake, physical activity, pharmacotherapy, and intercurrent illness, all of which may influence body composition trajectories (20, 21). Fifth, although MFR was deliberately excluded from the regression model to avoid the most overt form of circular inference, BMI remains mathematically and biologically related to body fat percentage, which itself constitutes a core component of the operational SO definition. The BMI–SO association reported here therefore partly reflects this intrinsic conceptual overlap rather than a wholly independent aetiological relationship, and BMI is best interpreted as a clinically accessible screening surrogate for the underlying compositional phenotype. Likewise, the conditional sex coefficient should not be interpreted as evidence of an independent population-level risk, given that the marginal sex-specific prevalence is, by design of the sex-specific thresholds, virtually identical. Finally, the longitudinal findings represent observed reclassification under irregular follow-up intervals and should not be interpreted as formal disease incidence.

## Conclusion

Sarcopenic obesity affects approximately one in four obese Malaysian adults when defined using the present body composition-based adapted ESPEN/EASO framework; this estimate is intrinsically tied to the operational definition adopted (sex-specific body fat percentage thresholds combined with population-derived lowest-quartile MFR cut-offs) and should be interpreted as the proportion of the obese population meeting those compositional criteria, not as an externally validated epidemiological prevalence. Skeletal muscle mass index-based criteria are fundamentally inadequate for SO detection in obese populations. BMI is the strongest statistical correlate of the body composition-based SO phenotype in our conditional model, although this association should be interpreted with caution given the partial conceptual overlap between BMI and the adiposity component of the operational definition; baseline BMI is the only independent predictor of longitudinal reclassification to SO in Cox proportional hazards regression, with body fat percentage losing independent significance once BMI is included, reflecting the well-recognised collinearity of these adiposity measures. Longitudinal BIA monitoring is clinically warranted in obese patients, as a meaningful proportion undergoes body composition reclassification within 12 months. Future studies should incorporate metabolic biomarkers, functional muscle assessments, and externally validated MFR thresholds to comprehensively characterise SO in Asian obese populations.

## Data Availability

The original contributions presented in the study are included in the article/Supplementary material, further inquiries can be directed to the corresponding author.
