# Supplementary material for: Prevalence and longitudinal development of sarcopenic obesity in an Asian obese population: a large bioelectrical impedance analysis study
Source: Front Nutr. 2026 Jun 2;13:1828960. doi: 10.3389/fnut.2026.1828960 (PMC13268986; doi:10.3389/fnut.2026.1828960)
Supplement: Supplementary file 1 [file Table_1.DOCX]

**Supplementary Material**

**Supplementary Table. Nested Cox proportional hazards regression models for time to reclassification to sarcopenic obesity.**

Three nested Cox proportional hazards models were fitted to the longitudinal cohort (n = 724 initially non-sarcopenic obese individuals with at least one follow-up bioelectrical impedance assessment; 48 events). Time to event was defined as time from baseline to first observed reclassification to sarcopenic obesity, with right-censoring at last available follow-up for participants without an event. Confidence intervals were derived using non-parametric bootstrap resampling (500 replicates) to provide stable interval estimates given heterogeneous follow-up intervals.

| **Variable** | **HR** | **95% CI (bootstrap)** | **p-value** | **Comment** |
| --- | --- | --- | --- | --- |
| ***Model A: age + sex + BMI (body fat % not included; n = 724, events = 48)*** | | | | |
| Age (per year) | 1.011 | 0.978–1.043 | 0.424 | *Not significant* |
| Sex (male) | 1.068 | 0.415–1.989 | 0.859 | *Not significant* |
| BMI (per 1 kg/m²) | **1.133** | **1.072–1.209** | **<0.001** | *Significant* |
| ***Model B: age + sex + body fat percentage (BMI not included; n = 724, events = 48)*** | | | | |
| Age (per year) | 1.002 | 0.972–1.033 | 0.861 | *Not significant* |
| Sex (male) | 4.155 | 1.217–15.525 | 0.014 | *Significant when BMI excluded* |
| Body fat % (per 1%) | **1.151** | **1.073–1.271** | **<0.001** | *Significant* |
| ***Model C: full model (age + sex + BMI + body fat %; this is Table 4 in main text; n = 724, events = 48)*** | | | | |
| Age (per year) | 1.008 | 0.976–1.039 | 0.538 | *Not significant* |
| Sex (male) | 1.979 | 0.470–8.307 | 0.293 | *Not significant* |
| BMI (per 1 kg/m²) | **1.098** | **1.019–1.195** | **0.012** | *Significant* |
| Body fat % (per 1%) | 1.059 | 0.962–1.185 | 0.249 | *Lost significance vs Model B (collinearity)* |

*Bold entries denote statistically significant predictors (p < 0.05). HR, hazard ratio; CI, confidence interval. Models A and B are sensitivity analyses to support the collinearity interpretation of Model C (the full model presented as Table 4 in the main text). The Pearson correlation between baseline BMI and baseline body fat percentage in the longitudinal cohort was r = 0.58 overall (r = 0.61 within each sex), which accounts for the loss of independent significance of body fat percentage in Model C despite its strong univariable association with reclassification in Model B.*

All models satisfied basic Cox PH assumptions (graphical inspection of log-minus-log survival curves did not suggest gross violation). Analyses were performed in a numerically stable Newton–Raphson implementation of the Cox partial-likelihood maximization with Breslow handling of ties; bootstrap resampling drew 500 replicates with replacement, refitting the full model on each replicate to obtain percentile-based 95% confidence intervals.
